# Endotracheal intubation with airtraq® versus storz® videolaryngoscope in children younger than two years - a randomized pilot-study

**DOI:** 10.1186/1471-2253-12-7

**Published:** 2012-04-30

**Authors:** Martin Kryspin Sørensen, Rolf Holm-Knudsen

**Affiliations:** 1 Department of Anaesthesia, Centre of Head and Orthopaedics, Copenhagen University Hospital, Rigshospitalet, Blegdamsvej 9, 2100, Copenhagen, Denmark; 2 Department of Anaesthesia, Centre of Head and Orthopaedics, Copenhagen University Hospital, Rigshospitalet, Blegdamsvej 9, 2100, Copenhagen, Denmark

**Keywords:** General anaesthesia, Airway management, Intratracheal intubation

## Abstract

**Background:**

New laryngoscopes have become available for use in small children. The aim of the study was to compare the Storz® videolaryngoscope (SVL) to the Airtraq® Optical laryngoscope (AOL) for tracheal intubation in children younger than two years of age who had a normal airway assessment. Our hypothesis was that the SVL would have a better success rate than the AOL.

**Methods:**

Ten children aged 2 years or younger scheduled for elective cleft lip/palate surgery were included. The anesthesia was standardized and a Cormack-Lehane (CL)-score was obtained using a Macintosh laryngoscope. After randomization CL-score and endotracheal tube positioning in front of the glottis was performed with one device, followed by the same procedure and intubation with the other device. The video-feed was recorded along with real-time audio. The primary endpoint was the success rate, defined as intubation in first attempt. Secondary endpoints were the time from start of laryngoscopy to CL-score, tube positioning in front of the glottis, and intubation.

**Results:**

Two intubation attempts were needed in two of five patients randomized to the SVL. The difference in time (SVL vs. AOL) to CL-score was 4.5 sec (p = 0.0449). The difference in time (SVL vs. AOL) to tube positioning was 11.6 sec (p = 0.0015). Time to intubation was 29.0 sec for SVL and 15.8 sec for AOL.

**Conclusion:**

No difference in the success rate of endotracheal intubation could be established in this ten patient sample of children younger than two years with a normal airway assessment scheduled for elective cleft lip/palate surgery. However, the Airtraq® Optical videolaryngoscope showed a number of time related advantages over the Storz® videolaryngoscope. Because of the small sample size a larger trial is needed to confirm these findings. Both devices were considered safe in all intubations.

**Trial registration:**

ClinicalTrials.gov; Identifier NCT01090726.

## Background

Videolaryngoscopes and optical laryngoscopes have been developed and used in adults during the last decade, and some of these are now available for small children [1]. The devices have proven effective in adults [2], but whether downscaling the adult equipment will work efficiently in small children is yet to be proven, though good initial experiences are reported [3]. Small children are not only shorter than larger children and adults, but relative differences in the dimensions of the mouth and of the upper airways are present between the groups. Small children have a relatively larger tongue that takes up more space in the oral cavity. The jaw is shorter making it more difficult to displace the tongue, and the larynx is localized more cephalad. The epiglottis is long, narrow, omega-shaped, and angled towards the lumen of the airway [4]. For these reasons one cannot expect to have the same excellent overview in a small child with a downscaled laryngoscope blade per se as in a larger child or in an adult. Airway related problems are among the most critical complications in pediatric anesthesia, with an incidence four times higher among children aged one year or younger than in older children. Thus airway management in small children remains an important research area [5].

**Figure 1:**
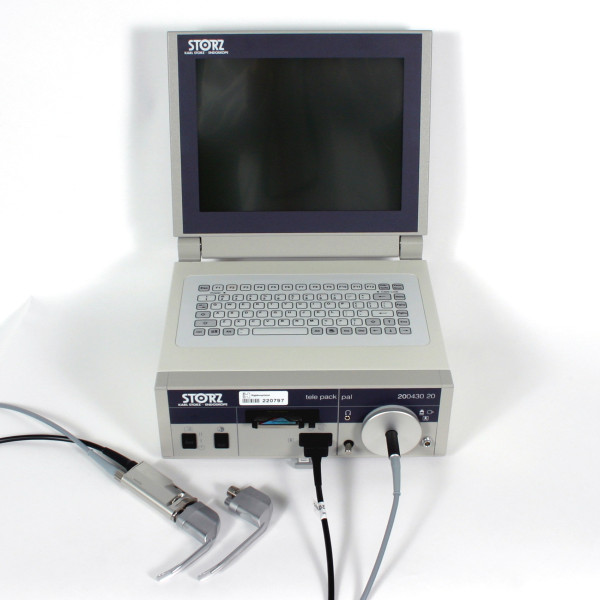
Storz® Berci-Kaplan videolaryngoscope.

**Figure 2:**
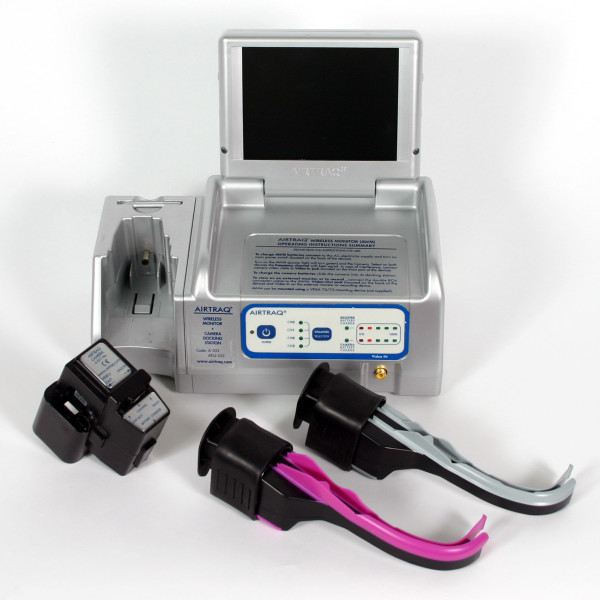
Airtraq® Optical videolaryngoscope.

The main objective of the pilot-study was to make a direct comparison of the Storz® Berci-Kaplan videolaryngoscope (SVL) (Figure 1) and the Airtraq® Optical videolaryngoscope (AOL) (Figure 2) using a Macintosh laryngoscope as reference for endotracheal intubation in small children with a normal preoperative airway assessment, thereby generating data that can be used for sample size calculations for larger trials in similar settings. The evaluation was focused on key components in the intubation procedure. Our hypothesis was that SVL would have a better success rate than AOL.

## Methods

The Regional Ethics Committee Capital Region (Address: Kongens Vænge 2, 3400 Hillerød, Denmark) approved the trial the 24^th^ of February 2010 (Protocol: H-2-2009-148). The trial was registered at ClinicalTrials.gov (Identifier: NCT01090726) prior to inclusion of the first patient. Written informed consent was obtained from the parents of all participating patients.

Patients were eligible if they were between 1 month and 2 years of age, were cleft lip/palate surgical patients with an ASA Physical Scale class 1–2, and had an indication of intubation for general anesthesia. We excluded patients if a history of difficult intubation was present, if the child had breathing difficulties during sleep or a dysmorphic feature with apparent micro- or retrognathia.

Patients were randomized 1:1 according to a computer generated list (GraphPad Software®, Inc., La Jolla, California, USA). A total of 10 envelopes were prepared for the trial, by staff with no other involvement in the trial. The patients were randomized to be intubated by either the SVL or the AOL.

The patients were monitored with a three lead ECG, non-invasive blood pressure measurement, pulse oximetry, capnography and temperature. Induction of anesthesia was done with sevoflurane and an intravenous access was established. Intravenous fentanyl (2.5 microgram/kg) and cisatracurium (0.1 mg/kg) was given. Mask ventilation and sevoflurane inhalation were continued for 4 minutes after which the time measurements were done and the endotracheal intubation was performed. Intubation was preceded by a crossover design with three laryngoscopies performed with three different laryngoscopes. Between each laryngoscopy the patient was ventilated by face mask. First, laryngoscopy was performed with a Macintosh blade and Cormack-Lehane (CL)-score [6] and timing to CL-score were registered. Then laryngoscopy was performed with either AOL or SVL depending on randomization and the CL-score with this device was registered. Time to CL-score and time to “ready to intubate” position of the endotracheal tube just in front of the glottis were also registered. Finally the patient was intubated using the other device (AOL or SVL). The CL-score with this device was registered along with time to CL-score, time to position the endotracheal tube in “ready to intubate” in front of glottis and time to complete endotracheal intubation, defined as passage of the vocal cords with the endotracheal tube. The evaluation of the airway was performed using a Macintosh blade, which is our standard method in children weighing more than 3 kg. A laryngoscope blade size 0 was used in children younger than six months otherwise a size 1 blade was used. When using the AOL the size was chosen from the recommendation on the package. AOL “infant” (size 0) for endotracheal tubes 3.0 and 3.5, and AOL “pediatric” (size 1) for size 4.0. With the SVL a “Miller-like” blade size 0 was used. The blade resembles an ordinary Miller blade, but is lower and half a cm longer. In all cases with all three devices the tip of the laryngoscope blade was placed in the vallecula and external pressure was applied to the neck if necessary. Preformed oral Microcuff ® endotracheal tubes were used for intubation in all cases. When using the SVL a malleable stylet with the tip bent as a hockey-stick was placed in the tube. Only indirect laryngoscopy, using the video monitor, was attempted with the SVL. When using the AOL only the distal half of the preformed tube was kept in the tube conduit during advancement of the tube as described by Xue et al [7]. The experiment was finished upon completed intubation, defined by verification of ETT placement. Only two intubation attempts were accepted. After this the experimental setup would be considered finished. In case of continued failure to intubate the responsible anesthesiologist would determine how to proceed. All investigations were performed by one experienced pediatric anesthesiologist, who had performed 20 or more endotracheal intubations with each of the new devices (AOL, SVL) in small children prior to inclusion of patients in the trial. Both the AOL and the SVL were connected via their monitors to a laptop computer. The video-feed was integrated in the software program Final Cut Pro 7 (Apple Inc., California, U.S.A.) with live audio from the room. An equipment rack was built for the purpose with monitors, laptop and cables securely clamped. This ensured that the setup would be the same for all patients. A switch made it possible to easily control the source of video-input to the computer, without affecting the ease of changing devices, the time recording, or the safety of the patient. The recording was started when the first laryngoscopy with the Macintosh laryngoscope was initiated. Time recording was done with both video and audio recording and by having the anesthesiologist mark the endpoints with a clear voice. The integrated recording was analyzed after the procedure. Endpoints were measured and evaluated independently two times, and then they were compared to identify potential discrepancies. If a discrepancy was found, the shortest time or best rating was chosen.

The primary endpoint was the success rate, defined as intubation in first attempt. Secondary endpoints were CL, time from start of laryngoscopy to CL, time to ETT positioning in front of the glottis, and time to endotracheal intubation. Time measurement was started from the point where the device was lifted off the table. Time to CL-score was recorded, and it was defined as the time point when the best possible view was achieved including the use of external manipulation. If a secondary intubation attempt was needed, all time recordings were based on the secondary attempt alone.

### Statistical analysis

No sample size calculation has been performed. An objective of this pilot-study was to determine the baseline data, on which a sample size calculation could be made for similar but larger studies.

The Wilcoxon test was used for statistical analysis of paired data. A P-value of less than 0.05 was considered statistical significant. All statistical calculations were done using SAS statistical software, Version 9.1 (SAS Institute, Cary, NC, USA).

## Results

**Figure 3:**
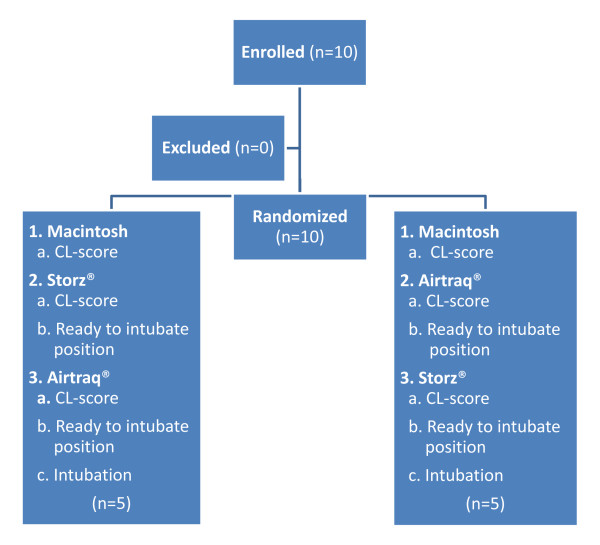
Trial profile.

In the period from March 2010 to October 2010 we included ten patients in the trial (Table 1; Figure 3). The SVL-group weighed 7.4 (1.1) kg and the AOL-group weighed 8.5 (2.9) kg. The age was 6.8 (5.4) months in the SVL-group and 8.4 (7.4) months in the AOL-group. Due to technical problems in two of five patients randomized to the SVL, two intubation attempts were needed: one due to fogging of the lens and the other due to sudden separation of the blade from the handle. All patients were intubated in the first attempt with the AOL (Table 2). The difference in time to CL-score between the devices (SVL-AOL) was 4.5 sec (p = 0.0449). The difference in time between the devices (SVL-AOL) to place the ETT in a “ready to intubate position” in front of the glottis was 11.6 sec (p = 0.0015) (Table 3). Time to endotracheal intubation was 29.0 sec for SVL and 15.8 sec for AOL. All patients had a CL-score of 1 with both the SVL and the AOL, whereas three patients had a CL-score of 2 with a Macintosh blade. No patient developed postoperatively stridor. No time discrepancy of more than 1 sec was found between the two independent evaluations of the integrated video and audio recordings.

**Table 1:** Patient characteristics, case by case

**Allocation**	**SVL**	**AOL**	**AOL**	**SVL**	**AOL**	**AOL**	**SVL**	**AOL**	**SVL**	**SVL**
Weight, kg	7.1	6.8	9.8	9.3	6.7	6.0	6.9	13.0	6.3	7.6
Age, months	7	3	16	16	3	3	3	17	4	4

**Table 2:** Results, case by case

**Case**	**1**	**2**	**3**	**4**	**5**	**6**	**7**	**8**	**9**	**10**	**Mean (SD)**
Allocation - intubation	SVL	AOL	AOL	SVL	AOL	AOL	SVL	AOL	SVL	SVL	
CL-score, Macintosh	1	2	1	2	1	2	1	1	1	1	
CL-score, Airtraq	1	1	1	1	1	1	1	1	1	1	
CL-score, SVL	1	1	1	1	1	1	1	1	1	1	
Time to CL-score, Macintosh, sec	6	12	6	12	9	16	8	5	7	11	9.2 (3.49)
Time to CL-score, Airtraq, sec	6	9	7	5	6	7	7	7	13	7	7.4 (2.22)
Time to CL-score, SVL, sec	9	24	5	6	7	9	9	20	24	6	11.9 (7.64)
Ready to intubate position,											
Airtraq, sec	16	16	19	14	20	13	26	11	25	15	17.5 (4.97)
Ready to intubate position,											
SVL, sec	34	37	22	19	31	19	28	37	40	24	29.1 (7.84)
Intubation, sec	34	16	19	19	20	13	28	11	40	24	
Intubation attempts	2	1	1	2	1	1	1	1	1	1	

**Table 3:** Results

	**SVL-AOL**	**P-value**
Difference in time to CL-score, sec	4.5 (1.9)	0.0449
Difference in time to” Ready to intubate position”, sec	11.6 (2.6)	0.0015
Paired data, presented as mean (SD).		

## Discussion

In two well-matched groups (Table 1) intubation was successful in the first attempt in 5/5 with the AOL and 3/5 with the SVL. The AOL was significantly faster than the SVL in all measured procedural elements of intubation in small children. CL-scores were 1 in all measurements with the two devices (Table 2). All patients were safely intubated.

A few limitations of the study must be mentioned. First, only one anesthesiologist performed all the investigational procedures and did this unblinded. Although experienced in intubation of this patient group with these devices, his personal skills could be reflected in the results of the investigation. In order to eliminate this bias the number of consultants performing the laryngoscopies would have to be increased. However, this would introduce other confounders such as the differing in learning curves with the devices and variable overall intubation experience in small children between the consultants. It was therefore decided to have only one consultant with extensive training in intubating small children with both devices, although unintentional biases of the intubator could have influenced the results*.* Blinding was not possible in this study. We compensated for these limitations by having every experimental procedure video recorded chronologically with integrated audio from the operating room, to make the results as objective as possible. The video evaluation was done independently twice. Maximum discrepancies of only one second between the recordings on all endpoints were found, and we consider this part of the study reproducible. Secondly, too few patients were included in order to do statistical analysis of unpaired data. However, this was a pilot-study and as such the data can still be used for sample size calculations in similar settings of future trials.

In a comparison of the Glidescope® and the SVL on a pediatric mannequin, time to endotracheal intubation was 19.9 (6.1) sec with the SVL under “normal conditions” [8]. We recorded a time to intubation with the SVL of 29.0 sec, with all measurements being 19 sec or above, but we only had five observations. Our results, although based on very few observations*,* are supported by Vlatten et al. who performed a randomized trial comparing the SVL to direct laryngoscopy in 56 children, aged 4 years or younger. They reported “time to intubation” to be 27 sec [9].

The application of results from mannequin studies to the clinic is becoming increasingly controversial, as it has proliferated without being followed by clinical trials on humans [10]. A central problem with mannequin studies is that one does not know exactly where the mannequins differ from the variety in the population. One could speculate that when intubating patients one is more cautious, and that this will prolong the endpoint of “time to intubation” as compared to the mannequin setting.

The AOL has been evaluated in a case series of 20 children aged 14 days to 9 years with both normal and difficult airways, and in conclusion the device was found safe and beneficial [11]. Although the case series had a wider age range than our pilot-study, these findings are in good accordance with ours. A couple of maneuvers have been proposed to facilitate intubation with AOL easier. When using a preformed endotracheal tube one can bypass the exoteric tube conduit of the AOL with the upper part of the tube. Other maneuvers have been proposed to facilitate successful intubation with the AOL when tube advancement is difficult [12]. These include the use of a stylet, an Endoflex-tube, or a fiberoptic bronchoscope. It is best to optimize the view before an intubation attempt.

In this report we present data from a direct comparison between the Airtraq® and the Storz® Berci-Kaplan videolaryngoscope in small children. In the narrow age group investigated, there are distinct features in the airway dimensions, where they differ from infants and older children. This could pose a challenge in the use of the two devices. It is not possible to extrapolate our results in children with normal airways to small children with difficult airways, as narrow space in the airway poses special challenges to the equipment. Failure to intubate these children with the AOL has been published [13]. Further studies, or case series, in small children with difficult airways are awaited before the usefulness of these devices in these children can be established.

A novel endpoint in the current study was the “ready to intubate” tube positioning. The introduction of this endpoint allowed the use of a paired statistical analysis on a procedural relevant endpoint without increasing the risk for the patients. However, this endpoint is a surrogate endpoint for intubation with limited clinical impact. In a similar but larger trial, we would therefore recommend the use of “time to intubation” or “success rate” in an unpaired design as primary endpoint with our data as the basis for the sample size calculation of a direct comparison between the Airtraq® and the Storz® Berci-Kaplan videolaryngoscope in small children.

In conclusion, no difference in the success rate of endotracheal intubation could be established in this ten patient sample of children younger than two years with a normal airway assessment scheduled for elective cleft lip/palate surgery. However, the Airtraq® Optical videolaryngoscope showed a number of time related advantages over the Storz® videolaryngoscope. Because of the small sample size a larger trial is needed to confirm these findings. Both devices were considered safe in all intubations.

## Competing interests

Both authors declare that they have no competing interests.

## Authors' contributions

MKS wrote the protocol, assisted in data collection, did the statistical analyses, and wrote the manuscript. RHK assisted in writing the protocol, collected the data, assisted in the statistical analyses, and assisted in writing the manuscript. Both authors read and approved the final manuscript.

## Pre-publication history

The pre-publication history for this paper can be accessed here:

http://www.biomedcentral.com/1471-2253/12/7/prepub
